# Examining Procrastination Across Multiple Goal Stages: A Longitudinal Study of Temporal Motivation Theory

**DOI:** 10.3389/fpsyg.2018.00327

**Published:** 2018-04-03

**Authors:** Piers Steel, Frode Svartdal, Tomas Thundiyil, Thomas Brothen

**Affiliations:** ^1^Human Resources & Organizational Behaviour, University of Calgary, Calgary, AB, Canada; ^2^Department of Psychology, UiT The Arctic University of Norway, Tromsø, Norway; ^3^Human Resources & Organizational Behaviour, Central Michigan University, Mount Pleasant, MI, United States; ^4^Department of Psychology, University of Minnesota, Minneapolis, MN, United States

**Keywords:** procrastination, temporal trajectories, motivation, self-regulation, longitudinal, pacing style

## Abstract

Procrastination is among the most common of motivational failures, putting off despite expecting to be worse off. We examine this dynamic phenomenon in a detailed and realistic longitudinal design (Study 1) as well as in a large correlational data set (*N* = 7400; Study 2). The results are largely consistent with temporal motivation theory. People’s pacing style reflects a hyperbolic curve, with the steepness of the curve predicted by self-reported procrastination. Procrastination is related to intention-action gaps, but not intentions. Procrastinators are susceptible to proximity of temptation and to the temporal separation between their intention and the planned act; the more distal, the greater the gap. Critical self-regulatory skills in explaining procrastination are attention control, energy regulation and automaticity, accounting for 74% of the variance. Future research using this design is recommended, as it provides an almost ideal blend of realism and detailed longitudinal assessment.

## Introduction

Procrastination is inherently dysfunctional, having been linked to a variety of major problems, from national and consumer debt (Steel, 2011; Sunstein, 2011) to unemployment and job search (Lay and Brokenshire, 1997; Van Hooft et al., 2013) to workplace cyberslacking and presenteeism (O’Neill et al., 2014; Wan et al., 2014). Given the importance and prevalence to so many outcomes, there has been disproportionately little motivational research on procrastination, almost to the point of neglect (Van Eerde, 2000; Steel, 2007). This is consistent, however, with the neglect of time in the motivational field (e.g., Fried and Slowik, 2004; Locke and Latham, 2004; Sonnentag, 2012) and the study of procrastination requires making choices over time (i.e., putting off despite being worse off). Looking forward, we argue that to develop an understanding of motivational failures, it is important to address these gaps in research on procrastination (e.g., Steel, 2007) and time (e.g., Fried and Slowik, 2004; Lord et al., 2010). In this study, we examine how procrastination manifests over time, employing the lens of temporal motivation theory (Steel and König, 2006).

### Temporal Motivation Theory

Described as one of the more comprehensive and promising theories equipped to further our understanding of procrastination is Steel and König’s (2006) temporal motivation theory (Lord et al., 2010; Schmidt et al., 2013). Temporal motivation theory is a meta-theory of motivation that integrates expectancy theory and hyperbolic discounting (from behavioral decision theory) with need theory and prospect theory (Hodgkinson and Healey, 2008). A simplified formulation of temporal motivation theory is:

Motivation =Expec⁢tan⁡cy×Value1+Im⁡pulsiveness×Delay

As per expectancy theory, motivation increases when people are confident of acquiring (i.e., expectancy) a desired reward or outcome (i.e., value). However, per behavioral decision theory and need theory, motivation is reduced when there is a large amount of time before the reward is realized (i.e., delay) and when we are sensitive to delays (i.e., impulsiveness). The constant “1” is added to prevent the equation approaching infinity when delay becomes effectively zero.

Temporal motivation theory, being a meta-theory, is explicitly designed to incorporate the empirically validated aspects of other formulations (Anderson, 2007; Schmidt et al., 2009; Lord et al., 2010). By incorporating expectancy theory, for example, it is inextricably linked to key motivational variables that include self-efficacy and achievement motivation (Klingsieck, 2013). Goal setting theory itself is largely derivable from temporal motivation theory (Gröpel and Steel, 2008), effectively connecting the inductive or descriptive approach of goal setting to other disciplines and lines of research. Demonstrating further integration, Gustavson et al. (2014) focused on temporal motivation theory during a twin study, one that revealed impulsiveness and procrastination are inseparable at a genotypic level. Interpreting these results, the authors suggested that temporal motivation theory is also consistent with evolutionary psychobiology and neurobiology accounts of motivation. The full theory is particularly useful to test deviations from the rational model, such as in the efficacy of long-term incentive plan where Pepper et al. (2013) found that the way “senior executives assess probabilities and value is significantly affected by risk aversion, time discounting and uncertainty aversion” (p. 48).

Given this broad integration of perspectives, temporal motivation theory can provide insights into how procrastination manifests across three goal phases of self-regulation: goal choice, goal pursuit and goal striving (e.g., Kanfer, 2012; Steel and Weinhardt, 2018). We start by examining *goal choice* (i.e., the process of selecting a goal) and *goal pursuit* (i.e., the initial steps taken toward accomplishing the goal; Oettingen et al., 2001). There are three main mechanisms (i.e., pacing style, intention-action gap, availability of temptation) that temporal motivation theory highlights are important for goal choice and pursuit and we develop three corresponding hypotheses. Then we examine *goal striving*, which refers to the process of putting forth effort for goal fulfillment with reflection and interplay between cognitive and behavioral processes. Similarly, we generate six hypotheses based on temporal motivation theory to better understand goal striving variables: energy regulation, automaticity, stimulus control, temptation attention control, goal attention control and fear of failure.

### Goal Choice and Goal Pursuit

Based on temporal motivation theory, there are several observable results that should reflect or affect the manner with which we pursue our goals (i.e., goal pursuit) and choose whether to abandon them altogether (i.e., goal choice). Under this theme, we suggest three goal pursuit and choice aspects: pacing style, intention-action gap and availability of temptation. In the following section, we discuss how these aspects are expected to operate from a temporal motivation perspective.

#### Pacing Style

Students are often faced with assessing their work process speed and how those processes are spaced out over time, collectively known as *pacing style* (Blount and Janicik, 2002). People differ in when they do their work, some doing the bulk of their work earlier and others later (Mitchell et al., 2008; Gevers et al., 2009). Temporal motivation theory suggests, based on temporal discounting, that self-regulatory failure is often caused by undue sensitivity to delay. Despite desires for motivation to arise earlier, motivation is contingent on a goal’s temporal distance, where motivation increases hyperbolically as the time to the deadline draws near. As such, we expect procrastinators struggle to focus their efforts on temporally distant reward and challenges (e.g., Gevers et al., 2009).

Although these propositions have not been directly examined in a field setting or over time, there is reason to believe we will see these results. For example, in a review of the experimental research on discounting, Green and Myerson (2004) found that pacing style typically follows a hyperbolic curve. Similarly, Howell et al. (2006) found, in the context of a classroom, that when each of 94 students returned a single assignment, in aggregate, the results formed a hyperbolic curve. Self-proclaimed procrastinators clustered around the deadline. In another classroom example, Schouwenburg and Groenewoud (2001), using retrospective self-reports from 305 students, also found hyperbolic discounting, with steeper curves for procrastinators. Therefore, taken together, we expect:

Hypothesis 1a: Goal pursuit should typically follow a hyperbolic curve.
Hypothesis 1b: The degree of curve should be predicted by the degree of procrastination.

#### Intention-Action Gap

One hindrance to student achievement is the failure to follow through on a given intention (Anderson and Merrett, 1997), known as the intention-action gap. Temporal motivation theory leads us to believe that the intention-action gap can best be understood through *preference reversal*, an individual’s willingness to make plans, only to reverse their plans before goal accomplishment. This can arise through temporal discounting. For example, the differences between 365 and 366 days is trivial, so we are unlikely to choose a moderately smaller reward in a year if an extra day’s wait will provide a substantially larger reward. However, as year’s end approaches, our preferences may reverse. One year from now, the choice will be between an immediate reward and waiting an entire day and individuals are more likely to change their minds and take the immediate reward now that there is a *relatively* large time difference (Frederick et al., 2002; Green and Myerson, 2004). Due to temporal discounting, we expect preference reversal to be pronounced for procrastinators.

Given that a central tenet to both goal setting theory (Locke and Latham, 2004) and temporal motivation theory is that goal proximity increases motivation, either theory could be used to predict the intention-action gap. However, in contrast to temporal motivation theory, goal setting theory attributes preference-reversal to “additional specific information” that is received during goal pursuit (Latham and Seijts, 1999, p. 422). Notably then, this provides an opportunity to test goal setting’s explanation against temporal motivation theory, that is whether the intention-action gap is due to intervening sources of additional information (i.e., goal setting theory) or whether it reflects temporal discounting induced preference reversal. If temporal motivation theory provides the better account, we would expect that intention-action gaps would not only be associated with procrastination but that procrastinators’ intention-action gap would also be especially sensitive to whether the goals were proximal or distal.

We expect the temporal motivational theory to provide the best account. Although the proposition has not been examined directly, Steel et al. (2001) did examine the relationship between work intentions and actions over five time periods, finding that student procrastinators had the same work intentions as non-procrastinators but had difficulty acting upon them at the start of the course. Consistent with this failure to follow through, procrastinators typically agree with the statement “No matter how much I try, I still put things off” (Stainton, 1993, Unpublished). However, toward the end of the course, Steel et al. noted that this pattern reversed and procrastinators ended up doing more work than they originally intended. Therefore, we predict:

Hypothesis 2a: Procrastination will be positively related to intention-action gaps but not related to intentions.
Hypothesis 2b: The positive relationship between intention-action gaps and procrastination will be larger for distal goals than proximal goal.

#### Proximity to Temptation

One clear driver of self-regulatory failure is the environment (Nguyen et al., 2013; Parker et al., 2014), especially the proximity an individual has to temptation. People procrastinate less when a temptation is further away (Lord et al., 2010), consistent with temporal motivation theory. For example, Lord et al. suggest that background temptations, such as the internet and email, sometimes possess greater utility than organizationally important activities. That is, at least, until the deadlines near for work projects.

Consequently, the more proximal the temptation, the greater should be the procrastination. Although we are unaware of a study that directly examines this proposition in either a field setting or in the context of performance, there is reason to believe this proposition will hold. In three experiments examining the self-regulation of eating, Vohs and Heatherton (2000) found that proximity to temptation increased the level of temptation. Furthermore, when individuals distanced themselves from the temptation, they were less likely to indulge in the temptation. Similarly, although not a focal part of their study, Fishbach and Shah (2006) examined students in the context of temptation with academic versus non-academic activities. In these studies, the removal or distancing from temptation decreased the likelihood of participating in the temptation. Based on these results, we expect:

Hypothesis 3: Temptation proximity will share a positive relationship with procrastination.

### Goal Striving

In addition to goal choice and pursuit, temporal motivation theory can help integrate our understanding of goal striving. To differentiate pursuit and striving, in her review of several models of goal phases, Kanfer (2012) notes that goal pursuit resides more in the planning of action and when behavior is initiated. In contrast, goal striving refers to actively putting forth effort toward an existing goal. In order to assist in goal striving, temporal motivation theory indicates several self-regulatory techniques should reduce the amount of irrational delay or procrastination, namely: energy regulation, automaticity, stimulus control, temptation attention control, goal attention control and fear of failure. Each of these are discussed in turn.

#### Energy

As Baumeister and Tierney (2011) review, willpower is exhaustible and, as individuals get more tired, they will more likely put things off. Many other scholars have made similar observations, from Freud (1923/1961) to Kuhl (2000). These researchers note that ego energy is used in many impulse restraining or delaying acts, such as thought suppression or even the act of volition. Unfortunately, this energy is quite limited, and as it depletes, the ability to self-control diminishes. From a temporal motivation theory perspective, there are two mechanisms at play. First, reduced willpower increases impulsiveness, making someone more vulnerable to temptations and preference reversal. Second, as we get tired, work becomes more aversive (i.e., decreasing in value), and it becomes more difficult to find sufficient motivation to continue striving (Steel, 2007).

Empirically, there is support for this view. Tiredness is one of the top three reasons students give for putting off work (Strongman and Burt, 2000), and approximately 28% of students indicated that lack of energy was a primary source for procrastination (Solomon and Rothblum, 1984; Peterson, 1987; Kachgal et al., 2001). Finally, Gröpel and Steel (2008) found a strong correlation of 0.60 between procrastination and energy level with a large diverse sample of 9,351 participants. Consistently, we expect that when individuals lose energy, or get more tired, they will be more likely to procrastinate. Therefore, we predict:

Hypothesis 4: Lack of energy will be positively related to procrastination.

#### Automaticity

Early theory suggests that increasing the number of choice points during goal striving can significantly increase the amount of procrastination, and the chance of self-regulatory failure that individuals experience (Silver, 1974). In support, several influential researchers argued that automaticity, a habitualized course of action that can be conducted with little or no conscious attention, is a powerful self-control technique (e.g., Karoly, 1993; Bargh and Barndollar, 1996). Automatic routines can take the form of planning or habits, and these automatic routines limit decision-making. Temporal motivation theory is a dynamic model of motivation dealing with choice among multiple options over time and suggests, due to preference reversal, that when individuals have routines, they will be less likely to procrastinate.

Although this proposition has not been examined directly in the context of a longitudinal field design, there is tangential support. Study habits are often linked to increased goal attainment and automaticity of routines (e.g., Bargh and Gollwitzer, 1994; Schmidt et al., 2013). Similarly, when individuals work to develop habits, they have been found to accomplish more and procrastinate less (Verplanken and Aarts, 1999). Therefore, we expect:

Hypothesis 5: Routines and planning will be negatively related to procrastination.

#### Stimulus Control

As mentioned above, when temptation is near, procrastination becomes likely. Akerlof (1991) theorizes that this increase in procrastination is due to *salience*, particularly regarding cues to do so. McCrea et al. (2008) found that our level of construal (i.e., whether we examine tasks in concrete or abstract fashion) affects procrastination. Incorporating these findings, Steel (2011) suggests, through temporal motivation theory, that impulsive choice is exacerbated by cues and stimuli. Consequently, *stimulus control*, which refers to surrounding oneself with task cues and eliminating signs that remind us of temptation, becomes a viable method to reduce procrastination. In other words, when we surround ourselves with task-relevant cues that confirm our mission and eliminate signs that remind us of temptation (e.g., indicators of new email), we will be less likely to procrastinate.

Though Steel and Klingsieck (2013) agree that stimulus control is a promising form of self-regulation, they admit relatively little direct study has been done on this in the context of procrastination. However, what is available is consistent with its efficacy as a self-regulatory technique. Ziesat et al. (1978) performed an experimental study, where college students were trained in stimulus control. The scholars found that students taught stimulus control studied for longer periods of time than the control. Another way to promote stimulus control is to improve the location where students study. Shoham-Salomon et al. (1989) and Mulry et al. (1994) instructed procrastinators to study in a specific location conducive to work, which was found to improve productivity. Therefore, we expect:

Hypothesis 6: Stimulus control will show a negative relationship with procrastination.

#### Temptation Attention Control

Consistent with the previous sections, temptation is often thought to be a cue for a self-regulatory breakdown, particularly when the individual is unable to stop thinking about the temptation (Mitchell et al., 2008). One way to alleviate this type of distraction occurs through temptation attention control (i.e., the diminution of attention toward a temptation). The self-regulatory mechanisms of temptation attention control are similar to automaticity and stimulus control, but instead focus on cognitive rather than physical attributes.

Temporal motivation theory would suggest that cognitively redirecting attention away from temptations should decrease procrastination (Steel, 2007). Mitchell et al. (2008) make a similar observation, “Interruptions or disruptive events like new tasks, phone calls, or visitors may also cause one to reassess the expected value of reaching a goal…. These interruptions often break individuals out of a script, take up time, and cause them to reassess their progress on their deadlines” (p. 210). Consequently, temptation attention control should reduce derailing from original plans or work scripts by providing fewer opportunities for procrastination.

There is there is reason to believe that we will find these results. Ainslie (1992) discusses how Freudian defense mechanisms (i.e., repression) provide much of their effectiveness by diverting “painful stimuli away from both awareness and motor responsiveness into the unconscious” (p. 128). Similarly, Mischel et al. (1989) famously examined delay of gratification in children and found that attentional tactics were effectively employed to prevent giving in to the smaller but more immediate temptation. Therefore, we expect:

Hypothesis 7: Temptation attention control will be negatively related to procrastination.

#### Goal Attention Control

Another powerful attentional control strategy that can decrease self-regulatory failure is *goal attention control*, where we not only focus our attention away from the source of temptation but toward other, less distracting options. As Mischel (1996) notes, focusing on images (i.e., arousing thoughts) other than the temptations are especially useful because they make fine distractions for tolerating delay. In addition, temporal motivation theory would suggest, anything that settles attention either on the positive aspects or away from negative aspects of a task can increase the value of the task, which would improve motivation.

However, there is a caveat to goal attention control. When focusing on the positive aspects of a goal, several researchers note that fantasy has the capacity to both hinder and help goal commitment (Ainslie, 1992; Gollwitzer, 1999; Oettingen, 2012). Unexpectedly, research suggests that a focus on the achievement of the goal itself decreases the likelihood of its completion. Temporal motivation theory would suggest that this occurs when individuals attend to symbolic images that give satisfaction, because individuals forgo their active pursuit.

Although we are unaware of research that has directly examined this proposition, there is reason to believe that individuals are capable of exploiting some form of this technique to curb self-regulatory problems (Siegel and Rachlin, 1995; Monterosso and Ainslie, 1999). For example, Morewedge et al. (2011), found that when individuals imagined consuming food, they reduced actual consumption. However, if we couple this idea of a positive goal fantasy with recognition of the present reality, goal achievement is significantly improved. Oettingen (2012) discusses how this “gap reflection” (i.e., the gap between present reality and future aspiration) helps to enable several other self-control mechanisms. To begin with, gap reflection aids in the development of definite plans for goal achievement through goal setting. Also, gap reflection helps to create an “implemental mind-set,” to use Gollwitzer’s (1999) term. A characteristic of an implemental mind-set is optimism about goal feasibility, thus improving its expectancy. This mirrors work done on process and outcomes visualization (Pham and Taylor, 1999). The authors suggest that outcome visualization, fantasizing about a desired result, is less conducive to achievement than process visualization, reflecting on goal striving or the “how” of goal pursuit.

Together, these lines of research generate two hypotheses:

Hypothesis 8a: Goal attention control will be negatively related to procrastination.
Hypothesis 8b: Positive goal fantasies should decrease procrastination only in conjunction with attending to the present.

#### Fear of Failure

There is significant debate regarding the role of irrational beliefs, such as fear of failure, in creating procrastination. Clinical opinion and theory often insist that irrational beliefs are not just minor contributors but the *primary* cause of procrastination (Pychyl and Flett, 2012), and consistent with this theorizing, open response research finds that between 7 and 17% of people indicate fear of failure the primary reason for procrastination (Steel, 2007). However, these results conflict with meta-analytic summary of effect sizes.

Irrational beliefs actually have a low to nil correlation with procrastination and sometimes the relationship works in the *opposite* direction (Steel, 2007). Such results suggest that perfectionism, an irrational belief, can prevent procrastination and its problems. Robert Slaney noted “perfectionists were *less* likely to procrastinate than non-perfectionists” (McGarvey, 1996). Alternatively, Rice et al. (2012) studied the issue using a longitudinal cross-panel design and concluded that the detrimental effects of procrastination only appear for non-perfectionists. Again, this suggests that perfectionism is actually protective.

What can explain this mismatch? Ferrari (1992) suggests that some irrational beliefs are adopted instrumentally, that is a form of self-deception used to protect self-esteem. Alternatively, fear of failure may feel like the source of procrastination, but it actually occurs in the presence of some other individual difference factors. Accordingly, Bui’s (2007) work suggests that procrastinators respond atypically and maladaptively to performance feedback: “low trait procrastinators are only motivated to work when there is a significant threat of evaluation. Conversely, this same level of threat appears to impair the high trait procrastinators” (p. 206). Also showing a differential response, König and Kleinmann (2005) found that non-procrastinators often sequence their work to start with the most onerous tasks, procrastinators tend to start with the most pleasurable tasks. More recently, Rosenbaum et al. (2014) termed this phenomenon “pre-crastination,” where some choose to do the worst thing first.

As a solution to the empirical inconsistency, temporal motivation theory offers a complementary explanation, suggesting that sensitivity to delay determines how people respond to stressful, aversive or anxious events. As Steel (2011) summarizes, “Impulsiveness also determines how we respond to task anxiety. For less impulsive individuals, anxiety is often an internal cue that motivates an early project start, but for more impulsive individuals it is a different story; anxiety over a deadline will lead straight to procrastination” (p. 13). Wanberg et al. (2010) work supports this interpretation. During goal pursuit, when less impulsive people perceive a setback or lack of progress, they typically responded with increased effort the next day. It was the opposite for impulsive people. Consequently, variables related to sensitivity to delay, such as impulsiveness or ego depletion, should explain the perceived relationship between fear of failure and procrastination.

Hypothesis 9: When people report fear of failure as reducing their motivation, its connection to procrastination can be accounted for by impulsiveness or a lack of energy.

## Study 1

### Method

#### Sample and Setting

Methodologically, Roe (2014) proposes an ideal design for motivation and performance studies. First and foremost, Roe suggests, they should not only use a longitudinal design, they should take a detailed approach. Though longitudinal studies are rare in motivational research, when they occur, they typically sample two or three randomly chosen time points. Roe argues that instead we should sample deeply across the entire duration of the task, favoring the number of observations over the number of the participants. This is particularly important in the examination of self-regulatory hypotheses, which by their nature investigates maintaining goal pursuit over time (Lord et al., 2010). Second, they should be gathered in the actual context. This is consistent with the push to use Natural Decision Making (NDM) settings (Klein, 2008). As Lipshitz et al. (2001) review, “NDM is an attempt to understand how people make decisions in real-world contexts that are meaningful and familiar to them” (p. 332). Third, data assessment should allow a bottom–up approach to capture individual temporal trajectories, ideally employing curve fitting methods that capture non-linear patterns. As Roe notes, exceedingly few studies have met any of these criteria, which he deems crucial to have a meaningful understanding of the “temporal footprint of work” (p. 101).

Using Roe’s (2014) suggestions for design, we used a computerized Personal System of Instruction (PSI). PSIs allow students to be frequently and accurately assessed over an extended period of time in a real but standardized setting. Consequently, we can study goal choice, pursuit and striving with large numbers of participants longitudinally through natural course progression. To this end, we used a 15-week introductory psychology course, similar to Steel et al. (2001). Participants were 171 undergraduate students, consisting of 40.6% males and 59.4% females. The course content was divided into 19 chapters, each consisting of Study Questions, Concept Previews, and Pretests, that student’s were expected to complete at their leisure, in preparation for a later supervised Progress Quiz. These four assignments of 19 each provided 76 time-points of assessment. At the end of the course, students completed a 77th assignment, the supervised final exam. Also of note, students could double the points they received for each of the 19 chapter Study Questions if they completed them by the recommended date, making them sensitive to procrastination. In general, coursework marks are ideal for assessing the effects of procrastination, substantively more than exam performance (Morris and Fritz, 2015). This study received ethics approval from the Institutional Review Board (IRB) at the University of Minnesota.

#### Assessment and Measures

Psychological testing was primarily computer administered and took place at three time periods: the first day of class, the last day of class, and immediately after the final exam. In addition to the computer-administered testing, students completed a printed inventory regarding their work intentions during the middle of the course. Finally, archival individual difference assessments were located and documented.

##### Self-report battery one

On day one of the course, students completed 116 items primarily assessing a variety of personality traits. This test battery was computer administered with 100% participant response. All items were assessed on a five-point scale, ranging from Strongly Agree to Strongly Disagree. We used the Irrational Procrastination Scale (IPS; Steel, 2010; Svartdal and Steel, 2017) to test trait procrastination. The IPS assesses procrastination as an irrational delay (i.e., “I delay tasks beyond what is reasonable”). Proximity to temptation was assessed with the content valid, six-item scale: *Temptation Susceptibility* (Steel, 2010). Sample items include “There are a lot of tempting diversions where I work” and “I could be doing something more enjoyable in an instant if I wasn’t working.”

Personality traits were measured using The Big Five Inventory (BFI; John et al., 1991), which is a 44-item personality measure assessing the big five traits. Given the overlap between conscientiousness and procrastination (Steel and Klingsieck, 2016), we use this to provide convergent validity as well as to test divergent or incremental validity. The two strongest predictors of performance are GMA and conscientiousness (Kuncel et al., 2004; Poropat, 2009) and being able to incrementally predict beyond them bolsters the importance of procrastination as a separate focus of investigation. To better explore the trait of conscientiousness, three of the NEO PI-R’s (Costa and McCrae, 1992) conscientiousness facet scales were employed: order, self-discipline, and achievement striving. Reflecting its close connection to procrastination, the self-discipline scale has several items that appear to be assessing the procrastination concept (i.e., “I waste a lot of time before settling down to work”) and should demonstrate the strongest associations (albeit negative) with procrastination. We employed the I^7^ scale (Eysenck et al., 1985), to measure impulsiveness, which assesses the broad understanding of the construct (Revelle, 1997).

##### Self-report battery two

At approximately mid-way through the course, students indicated their work intentions for the following week and the week after. The measure was administered through paper-and-pencil. It comprised of four items, two for each week: “How much work do you plan to do for this course?” and “How many quizzes do you plan to complete?” Participants indicated the exact hours and quizzes they had planned.

##### Self-report battery three

During the course’s last week, students completed a third test battery. To gain test-retest reliability, the IPS was administered again. Also, nine scales from Kuhl and Fuhrmann’s (1998) Volitional Components Inventory (VCI), version VCI-10-99, was adapted for use. The VCI divides itself into two categories of self-control, volitional inhibitions, and self-regulatory capability. Volitional inhibitions refer to reduced access to volitional competencies, that is, self-regulation and self-control. Typically, these symptoms show up under periods of frustration or stress. Two scales were used: *Lack of Energy* (originally labeled energy deficit) refers to volitional inhibition due to feeling dull or tired (e.g., “I feel zestless or drained when it comes to doing jobs I find objectionable”); *Attentional Distractibility*, which refers to difficulties in preventing attention wandering off goal (e.g., “I have a hard time concentrating”).

The VCI addresses the seven self-regulatory skills we examine. First, *Emotional Distractibility*, refers to difficulties in resisting temptation, except in this case where desire easily wanders *toward* other options (e.g., “I let myself get distracted by more pleasant things”). Second, *Conscious Attention Control* refers to mindfully keeping one’s attention focused that is purposefully putting one’s mind back toward the task (e.g., “I bring to mind again and again what I have to do”). Third, *Planning* refers to routinizing or scheduling the details of the task (e.g., “For my work, I try to keep a regular schedule”). Fourth, *Strategic Intention Control* refers to using external aids (e.g., to do lists) as a reminder about one’s intentions and provides a good measure of stimulus control. Fifth, *Emotional Perseverance Inhibition* refers to failure or fear of failure that results in “paralysis” or a loss of drive (e.g., “Fear of failure paralyzes me”). Sixth, *Implicit Attention Control* refers to the ability to automatically focus on the target task (e.g., “I find my attention riveted to what I am doing”). Finally, *Positive Goal Fantasy* refers to reflecting or imagining the reward that will accompany goal accomplishment (e.g., “I fantasize about how good it will feel to have achieved my goals”).

##### Self-report battery four

After the final exam, students evaluated the course and we asked a few additional questions, developing three course-specific measures composed of two items each. Course-specific refers to a state-level rather than a trait-level assessment that examines how the students behave during the last semester. The following three variables were assessed. First, we administered an additional procrastination measure (i.e., “In this course, how often did you put off school work more than was reasonable?”). Second, we assessed performance to student’s standard, supplementing objective academic performance (i.e., “How satisfied are you with the grade you expect to receive in this course?”). Third, we assessed another self-regulatory technique, *Study Routines*, which refers to the amount of effort put into developing regular study habits (i.e., “How much effort did you put into creating a weekly or daily study schedule”).

##### Archival data

General mental ability (GMA) was assessed through the American College Test (ACT) scores, used for college admissions. The overall composite score is the focus of this study as it gives a better gauge of GMA (ACT, 2014). Internal reliability of the ACT’s Composite score is.96 and it accounts for approximate 63% of the variance in high school GPA (ACT, 2014). ACT scores were available for 189 students or 87% of the sample.

### Results and Discussion

The reliabilities, means, standard deviations and bivariate correlations of collected self-report data are reported in **Table 1**. Before examining the hypotheses, we will examine the procrastination construct.

**Table 1:** Correlations among procrastination, GMA, personality, conscientiousness and self-regulatory variables.

	1	2	3	4	5	6	7	8	9	10	11	12	13
**Procrastination**											
1 Trait	*0.93*												
2 Course specific	**0.57**	*0.80*											
3 Observed	**0.41**	**0.61**	–										
**General mental ability**											
4 ACT: Comprehensive	0.01	0.02	0.09	–									
**Big Five Personality**											
5 Extraversion	-0.06	-0.02	0.05	-0.04	*0.83*								
6 Agreeableness	-0.05	0.13	0.12	-0.07	0.15	*0.72*							
7 Conscientiousness	**-0.45**	-0.13	-0.15	-0.14	0.08	**0.29**	*0.76*						
8 Neuroticism	0.10	0.08	0.02	0.02	**-0.32**	**-0.27**	**-0.27**	*0.83*					
9 Openness	-0.03	-0.02	0.07	-0.07	0.17	0.08	0.03	-0.16	*0.76*				
**Conscientiousness related**											
10 Impulsiveness	**0.27**	0.12	0.14	0.08	0.09	**-0.40**	**-0.41**	**0.21**	0.08	*0.79*			
11 Need for order	**-0.35**	-0.10	-0.01	-0.15	0.05	0.04	**0.62**	-0.04	0.00	**-0.33**	*0.68*		
12 Self-discipline	**-0.61**	**-0.33**	**-0.21**	-0.10	0.12	0.17	**0.71**	**-0.27**	0.03	**-0.35**	**0.52**	*0.79*	
13 Achievement striving	**-0.34**	-0.11	0.02	-0.11	**0.18**	**0.21**	**0.62**	**-0.23**	0.04	**-0.36**	**0.40**	**0.67**	*0.78*
14 Temptation susceptibility	**0.38**	0.17	0.06	0.14	0.12	0.02	**-0.39**	0.12	0.00	**0.23**	**-0.27**	**-0.47**	**-0.33**
**Self-regulation related**											
15 Attentional distract	**0.71**	**0.33**	**0.30**	-0.12	-0.09	-0.11	**-0.48**	**0.29**	0.00	**0.29**	**-0.31**	**-0.49**	**-0.27**
16 Conscious attention control	**-0.24**	-0.18	-0.11	-0.09	0.08	-0.01	0.21	0.04	0.10	0.04	**0.21**	**0.29**	**0.32**
17 Emotional distractibility	**0.75**	**0.35**	**0.26**	-0.08	-0.06	-0.10	**-0.40**	**0.21**	0.05	**0.39**	**-0.25**	**-0.50**	**-0.27**
18 Emotional perseverance inhibition	**0.33**	0.16	0.10	**-0.21**	-0.16	-0.10	-0.16	**0.54**	-0.03	0.10	0.05	**-0.30**	-0.09
19 Implicit attention control	**-0.63**	**-0.43**	**-0.27**	-0.18	0.10	0.12	**0.39**	-0.14	0.15	-0.14	**0.25**	**0.47**	**0.37**
20 Lack of energy	**0.64**	**0.32**	**0.24**	-0.06	-0.16	-0.11	**-0.36**	**0.33**	0.04	**0.26**	-0.20	**-0.45**	**-0.25**
21 Planning	**-0.52**	**-0.35**	**-0.21**	-0.20	0.03	0.02	**0.31**	0.06	0.10	-0.12	**0.36**	**0.47**	**0.37**
22 Positive goal fantasy	-0.15	-0.18	-0.08	-0.11	0.15	0.05	0.16	-0.03	0.17	0.03	0.17	**0.23**	**0.28**
23 Strategic intention control	**-0.26**	-0.13	-0.05	**-0.23**	**0.31**	0.05	**0.21**	0.10	0.12	-0.07	**0.35**	**0.23**	**0.27**
24 Study routine	**-0.35**	**-0.35**	**-0.21**	**-0.28**	0.04	0.11	**0.20**	0.09	-0.01	**-0.20**	**0.28**	**0.31**	**0.31**
Mean	3.1	2.6	–	21	3.6	4.0	3.6	2.7	3.6	2.9	3.2	3.5	3.6
Standard deviation	0.97	0.93	–	3.5	0.76	0.55	0.83	0.83	0.60	0.55	0.65	0.72	0.70

	14	15	16	17	18	19	20	21	22	23	24

**Procrastination**											
1 Trait											
2 Course specific											
3 Observed											
**General mental ability**											
4 ACT: Comprehensive											
**Big Five Personality**											
5 Extraversion											
6 Agreeableness											
7 Conscientiousness											
8 Neuroticism											
9 Openness											
**Conscientiousness related**											
10 Impulsiveness											
11 Need for order											
12 Self-discipline											
13 Achievement striving											
14 Temptation susceptibility	*0.72*										
**Self-regulation related**											
15 Attentional distractibility.	**0.39**	*0.91*									
16 Conscious attention control	-0.14	-0.12	*0.68*								
17 Emotional distractibility	**0.38**	**0.80**	0.00	*0.88*							
18 Emotional perseverance inhibition	0.18	**0.49**	0.02	**0.45**	*0.91*						
19 Implicit attention control	**-0.31**	**-0.54**	**0.46**	**-0.44**	-0.18	*0.71*					
20 Lack of energy	**0.32**	**0.63**	0.03	**0.72**	**0.50**	**-0.39**	*0.83*				
21 Planning	**-0.32**	**-0.31**	**0.34**	**-0.33**	0.01	**0.57**	**-0.24**	*0.83*			
22 Positive goal fantasy.	-0.08	-0.05	**0.31**	0.00	-0.06	**0.37**	-0.04	**0.39**	*0.81*		
23 Strategic intention control.	-0.09	-0.16	0.09	-0.17	0.07	**0.37**	-0.20	**0.48**	**0.38**	*0.76*	
24 Study routine	-0.06	-0.20	**0.23**	-0.16	0.14	**0.34**	-0.08	**0.49**	**0.30**	**0.39**	*0.59*
Mean	3.7	3.4	3.8	3.4	2.5	3.2	3.5	3.7	4.0	3.4	2.9
Standard deviation	0.73	0.81	0.48	0.82	1.0	0.63	0.81	0.85	0.71	0.88	0.76


*Results significant at *p* < 0.01 bolded. Cronbach alpha italicized along the diagonal.*

#### The Procrastination Construct

**FIGURE 1:**
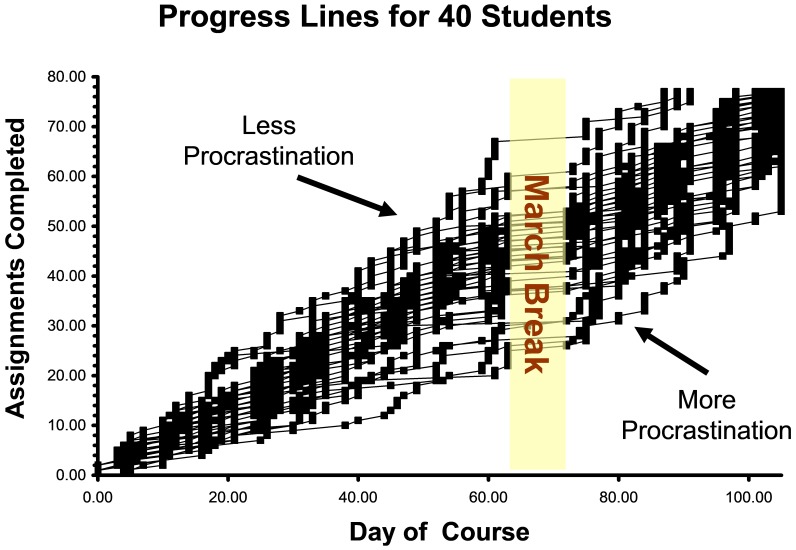
Pacing style of a representative student subsample completing 77 course assignments over a semester.

We measured procrastination three ways: a trait level, a state or course-specific level, and finally with observed behavior. To develop an observed measure of procrastination, we summarized student responses over the semester. We measured their progress at 77 different time points, as shown in **Figure 1** for a representative subsample (i.e., 40 students, which allows for the display of individual trendlines).

To model this information, we used area under the curve as the summary index; which has been considered a significant improvement over previous summary methods (e.g., Steel et al., 2001; Howell et al., 2006). Numerical integration by parts was employed to measure area under the curve. Non-procrastinating students who complete all assignments early show a progress line that approaches the maximum level (i.e., 77) and then plateaus until the end of the course and their observed area would be large. Procrastinating students who complete their assignments late should reach the maximum level only toward the end of the course. The observed area should be small. To make the index consistent in direction with other measures of procrastination (i.e., higher equates to more), all scores were multiplied by minus one. The inclusion of an observed measure of procrastination is notable here as this topic is almost exclusively based on self-reports, with a few exceptions (e.g., Elvers et al., 2003). Partly, this reflects that observed delay, as we obtained, may or may not be an irrational delay depending upon the respondents’ aspirations and priorities. Though we suspect that in this venue most delay will be irrational (i.e., PSI courses tend to be rife with procrastination), we need to confirm this.

The convergent validity among the procrastination measures are shown in the first three rows of **Table 1**. There is strong agreement between course specific and observed procrastination (*r* = 0.61), indicating that students are fairly honest and accurate assessors of their own procrastination and that observed delay, in this instance, is a decent proxy for irrational delay. Trait procrastination also had a strong correlation with course specific (*r* = 0.57) and observed procrastination (*r* = 0.41) as well as with other related measures. Trait procrastination correlated with conscientiousness and other conscientiousness related traits (i.e., Impulsiveness, Need for Order, Self-Discipline, Achievement Striving and Temptation Susceptibility) at an absolute average of 0.40. As expected, Self-Discipline had the highest correlation at -0.61. Showing divergent validity, correlations with other factors from the Big Five was an average absolute of 0.06, reaching a high of 0.10 with neuroticism. Furthermore, when trait procrastination from Self-Report Battery Three was compared with the Self-Report Battery One administration, a hiatus of approximately 4 months, test-retest was high (*r* = 0.67), indicating consistency of responding. We used the second administration of procrastination, which had slightly more respondents, for our analyses.

Finally, to emphasize procrastination as a relevant and distinct construct, we examined whether it incrementally predicts performance above GMA and conscientiousness, the strongest predictors of performance (Kuncel et al., 2004; Poropat, 2009). For trait and course-specific procrastination, we tested a broad battery of performance criteria: Study Question Average Grade, Total Average Grade, Total Assignments Completed, Final Exam, Course Letter Grade, and Performance to Own Standard. **Table 2** examines the incremental variance accounted for by trait and course-specific procrastination above GMA and conscientiousness together.

**Table 2:** Hierarchical regression analyses for determining the incremental predictiveness of trait and course specific procrastination above GMA and conscientiousness for performance.

Model	Performance measure	Total *R*^2^	Δ*R*^2^	ß
***With trait procrastination entering second***				
Model 1	Study Question Average Grade	0.20^∗∗^	0.17^∗∗^	-0.46
Model 2	Total Average Grade	0.39^∗∗^	0.02^∗^	-0.14
Model 3	Total Assignments Completed	0.06^∗∗^	0.06^∗^	-0.27
Model 4	Final Exam	0.18^∗∗^	0.04^∗^	-0.23
Model 5	Course Letter Grade	0.26^∗∗^	0.06^∗∗^	-0.28
Model 6	Performance to Own Standard	0.13^∗∗^	0.04^∗^	-0.22
Average Incremental Variance			0.07	
***With course specific procrastination entering second***				
Model 1	Study Question Average Grade	0.31^∗∗^	0.27^∗∗^	-0.52
Model 2	Total Average Grade	0.44^∗∗^	0.11^∗∗^	-0.33
Model 3	Total Assignments Completed	0.16^∗∗^	0.14^∗∗^	-0.37
Model 4	Final Exam	0.46^∗∗^	0.05^∗∗^	-0.22
Model 5	Course Letter Grade	0.41^∗∗^	0.22^∗∗^	-0.47
Model 6	Performance to Own Standard	0.41^∗∗^	0.32^∗∗^	-0.57
Average Incremental Variance			0.19	


*^∗^*p* < 0.05, ^∗∗^*p* < 0.001.*

Especially at the course-specific level, procrastination substantively predicts performance above GMA and conscientiousness. These results indicate that even if people are not typically procrastinators, when they do irrationally delay, the detriment to performance is large. Still, as expected, there was variation among in susceptibility to procrastination among these performance measures, with Study Questions being particularly sensitive. However, the single largest effect of procrastination is with Performance to Own Standard. Though procrastination is of concern to educators, it appears to be quintessentially an irrational or self-defeating delay as it is most troubling to the perpetrators of procrastination themselves.

#### Hypothesis Testing

With observed, course specific and trait procrastination measures, we have a variety of criteria. As per the bandwidth-fidelity dilemma, we tend to get better associations when matching *course specific* procrastination to *course-specific* behavior, highlighting the impact of procrastination. However, results tend to be more generalizable at the trait level. Observed behavior is the best at actually predicting course performance but runs into the issue of criterion contamination, as delay influence grades inherently by course design. Consequently, we emphasize course specific and trait procrastination analyses.

##### Goal choice and goal pursuit

Hypothesis 1 states that goal pursuit will follow a hyperbolic curve (1a) and the degree of the curve will be predicted by the degree of procrastination (1b). **Table 3** reports multiple regression analysis of date and course-specific procrastination predicting daily assignments completed (accounting for 13% of the variance). Goal pursuit typically followed a hyperbolic curve, providing support for hypothesis 1a. Consistent with hypothesis 1b, the significant interaction term between date and procrastination indicates that procrastination decreased or increased the degree of the curve. That is, the constant represents the average number of daily assignments (i.e., 0.95). Date represents the number of days left in the course, so as the course closes, the fewer assignments are done (i.e., -3.76). Procrastinators tend to due fewer assignments per day (i.e., -0.16), except at the end due to the interaction (i.e., Course Specific Procrastination/Date), which being positive (i.e., 2.62), means that when date gets small toward the end of course, people higher on procrastination tend to work a lot more. As people identify themselves as procrastinators, the less work is done at the beginning and the more at the end. Those reporting essentially no procrastination will actually finish early, with the threshold defined from our date coefficients (i.e., 3.75-2.62), leaving no work for the final days. As the level of procrastination increases, going above 1.1 on our 5-point scale, a curve appears and then deepens. On the final day, maximal procrastinators are showing a very sharp curve, completing over nine assignments on average, which is eleven times the highest average daily output for non-procrastinators. We get this exact same pattern of results with trait procrastination, though stronger results are obtained matching course behavior with course procrastination.

**Table 3:** Regression analysis summary of date and course specific procrastination predicting daily assignments completed.

Variable	*B*	β	*t*	Sig.
*Constant*	-0.95		-34.85	0.000
1/Date	-3.76	-0.35	-17.11	0.000
Course specific procrastination	0–0.16	-0.12	-15.88	0.000
Course specific procrastination/Date	-2.62	-0.68	-32.87	0.000


*Date represents days remaining in course, meaning that as the course closes, 1/Date increases in size. Procrastination needs to 1.1 or lower on a five-point scale not to see some increased pace at course end.*

Hypothesis 2 states that procrastination is not related to intentions but shares a relationship with intention-action gap (2a) and that positive relationship between intention-action gap and procrastination is greater for distal goals than for proximal goals (2b). Based on data from the 90 students who completed this section of the study, correlations between trait or course-specific procrastination with work intentions ranged from a minimum of 0.09 to a maximum of 0.23, which is significant at *p* < 0.05. In short, these results indicate that procrastinators typically intend to work *as hard or even harder* than non-procrastinators, and their work delays appear to be unintentional. Also, to further confirm if procrastinators tend to have a larger intention-action gap, hierarchical multiple regression was employed and the results can be seen in **Table 4**. We would expect procrastination to incrementally predict the duration of work as well as the amount of work completed after first considering intended effort. That is, procrastinators would do less work even after controlling for intentions. As the results in **Table 4** indicate, procrastinators did substantively less work than intended, especially as measured by course-specific procrastination. This provides evidence for the relationship between procrastination and the intention-action gap, which supports hypothesis 2a. With hypothesis 2b, the trend should intensify when results from “The Next Week” are compared to that of “The Week After.” With the one exception of trait procrastination and hours worked, **Table 4** shows that this trend was consistently observed. For example, the relationship between course specific procrastination and quizzes substantively increases when intentions next week (*R*^2^ = 0.07, *p* < 0.05) are compared to intentions made for the week after (*R*^2^ = 0.30, *p* < 0.001). In short, procrastinators’ intention-action gap was more pronounced (*p* < 0.001) when the time lag was 2 weeks instead of 1 week into the future. With 7 out of 8 analyses operating as expected, hypothesis 2b was supported.

**Table 4:** Hierarchical regression analyses for determining the incremental predictiveness of self-report procrastination indices above work intentions for predicting observed behavior.

Model	Work intention	Adjusted *R*^2^	Adjusted Δ*R*^2^	*B*
***With trait procrastination entering second***				
Model 1	Hours Worked Next Week	0.16^∗∗^	0.03^∗∗^	-0.28^∗∗^
Model 2	Quizzes Completed Next Week	0.02^∗∗^	0.00^∗∗^	0.00^∗^
Model 3	Hours Worked Week After	0.06^∗∗^	0.06^∗∗^	-0.27^∗∗^
Model 4	Quizzes Completed Week After	0.09^∗∗^	0.09^∗∗^	-0.33^∗∗^
***With course specific procrastination entering second***				
Model 1	Hours Worked Next Week	0.17^∗∗^	0.05^∗∗^	-0.37^∗∗^
Model 2	Quizzes Completed Next Week	0.07^∗∗^	0.05^∗∗^	-0.24^∗∗^
Model 3	Hours Worked Week After	0.22^∗∗^	0.22^∗∗^	-0.51^∗∗^
Model 4	Quizzes Completed Week After	0.30^∗∗^	0.30^∗∗^	-0.62^∗∗^


*^∗^*p* < 0.05, ^∗∗^*p* < 0.001.*

Hypothesis 3 states that there is a positive relationship between temptation proximity and procrastination. To examine this effect, we first examined the correlation between the Temptation Susceptibility scale and the procrastination indices, as reported in **Table 1**. At a trait level, the relationship is significant, moderate in strength (*r* = 0.38) and in the expected, positive direction. However, proximity to temptation does not appear to be entirely assessing environmental conditions. People who score highly on the Temptation scale also scored higher on VCI’s *Emotional* and *Attentional Distractibility* scales. In fact, *post hoc* analyses show that Temptation fails to incrementally predict procrastination above these two variables [Δ*R*^2^ = 0.01, *F*(1,163) = 2.2, *p* = 0.14]. Consequently, proximity to temptation, an environmental variable, is confounded with personal susceptibility to temptation, an individual difference variable. As might be expected, people who say they work near temptation are likely those who find a disproportionate percentage of life’s pleasures very tempting themselves.

##### Goal striving

Hypothesis 4 predicts that there will be a negative relationship between energy level and procrastination. As seen in **Table 1**, VCI’s Lack of Energy scale shares a moderate to strong significant relationship with all of the procrastination indices (trait: *r* = 0.64; course-specific: *r* = 0.32; observed *r* = 0.24).

Hypothesis 5 states that the use of routines and habits will show a negative relationship with procrastination. Automaticity was assessed at a trait level with VCI’s Planning and at the course-specific level with Study Routines, with the trait and course-specific scores correlating together at 0.49. Both Planning (trait: *r* = -0.52; course-specific: *r* = -0.35; observed *r* = -0.21) and Study Routines (trait: *r* = -0.35; course-specific: *r* = -0.35; observed *r* = -0.21) were consistently and negatively associated with the procrastination indices.

Hypothesis 6 states that stimulus control shows a negative relationship with procrastination. As per **Table 1**, this received mixed support as trait procrastination shared a moderate negative significant relationship with VCI’s Strategic Intention Control (*r* = -0.26). On the other hand, though both course-specific and observed procrastination were negative, neither were significant.

Hypothesis 7 predicts that temptation attention control will show a negative relationship with procrastination. We operationalized temptation attention control utilizing two different VCI scales: Attentional Distractibility and Emotional Distractibility. Both scales deal with assessing the degree people are able to suppress other needs and desires. As per **Table 1**, the bivariate analysis indicates that both facets of temptation attention control are strongly related to procrastination, with Emotional Distractibility reaches a correlation of 0.75 with trait procrastination. The combined contribution of both scales was an adjusted *R*^2^ of 0.59 (*p* < 0.001).

Hypotheses 8 examines goal attention control, with 8a predicting that there will be a negative relationship between goal attention control and procrastination. Goal attention control was measured using two VCI scales, Conscious Attention Control and Implicit Attention Control. As per **Table 1**, Conscious Attention Control shared a weak-moderate relationship with procrastination, typically correlating around -0.20. Implicit Attention Control, however, correlated much more strongly, as high as -0.63 with trait procrastination. This difference in association appears to reflect that Conscious Attention Control refers to *attempting* to focus one’s attention, while Implicit Attention Control refers to *automatic* success. Notably, given the similarities between goal attention control and temptation attention control, we conducted a supplementary analysis to determine if each represented separate pathways to reduce procrastination and accounted for independent variance. Despite both focusing on attention, multiple regression analysis indicated each separately predicts: Conscious Attention Control (β = -0.18, *p* < 0.001), Attentional Distractibility (β = 0.28, *p* = 0.001) and Emotional Distractibility (β = 0.51, *p* < 0.001). Following up on this, we then added all our other previous significant predictors. Reaching a *R*^2^ of 0.74, all three incrementally predicted: Lack of Energy (β = 0.23, *p* < 0.001), Planning (β = -0.19, *p* = 0.002), and Study Routines (β = -0.13, *p* = 0.01).

Hypothesis 8b focuses on the use of fantasy and whether it can reduce procrastination. Using VCI’s Positive Goal Fantasy, as expected, it alone was not significantly correlated with any of the procrastination indices. However, it should interact with goal attention control, which we measured with Implicit Attention Control. Of note, detecting interaction effects in field studies is notoriously difficult (McClelland and Judd, 1993). Given our sample size, this typically requires a large effect to detect as a moderate interaction effect of Δ*R*^2^ = 0.02 (Evans, 1985) provides a statistical power of only approximately 0.40. Using hierarchical regression, allowing the VCI measures to enter first, followed by the interaction term, the interaction was not statistically significant using trait level procrastination. On the other hand, the interaction term was significant for both course specific [Δ*R*^2^ = 0.04, *F*(1,148) = 6.95, *p* < 0.01] and observed procrastination [Δ*R*^2^ = 0.04, *F*(1,149) = 7.05, *p* < 0.01]. Overall, hypothesis 8b is supported.

Hypothesis 9 predicted that when fear of failure is self-reported as reducing motivation, its connection to procrastination should be fully accounted for by impulsiveness and/or lack of energy. As per **Table 1**, VCI’s Emotional Inhibition scale has a moderate relationship with trait procrastination (*r* = 0.33), though negligible with course-specific or observed procrastination. Focusing on trait procrastination, hierarchical regression indicates that it adds no incremental variance above Lack of Energy and Impulsiveness [Δ*R*^2^ = 0.00, *F*(1,164) = 0.05, *p* = 0.83], with only Lack of Energy significantly predicting (*p* < 0.001). Consequently, though people may attribute fear of failure as a cause of their procrastination, it only appears to be the case when energy levels are low. Hypothesis 9 is supported.

## Study 2

Complementing Study 1, Study 2 verified the relationships among self-regulation related variables and trait procrastination, focusing on hypotheses 3–9. Here, we took a cross-sectional approach, allowing for far greater sample size and associated statistical power.

### Method

#### Sample and Setting

Data collection was part of a larger effort to determine the epidemiology of procrastination, as published by Steel and Ferrari (2013), which should be referenced for details regarding administration. For this analysis, 7400 online participants responded, who also provided basic demographic information. Of this group, 2435 indicated they were full-time students.

#### Assessment and Measures

Three key measures were employed. We administered the IPS for procrastination (Steel, 2010). To enable re-examination of hypotheses 3–9, we administered Kuhl and Fuhrmann’s (1998) VCI and the Temptation Susceptibility scale (Steel, 2010).

#### Statistical Analysis

The analytical approach was similar to that of Study 1, using correlations and regression to document predicted associations.

#### Results

Procrastination’s bivariate correlations with the VCI and Temptation Susceptibility scale are reported in **Table 5**. To avoid sample characteristics as being a possible confounding or moderator variable, we also report the results for the student sample.

**Table 5:** Bivariate correlations between procrastination and VCI scales as well as Temptation Susceptibility.

	Study 2	Study 2
	(Full)	(Student)
1 Temptation Susceptibility	0.52	0.53
15 Attentional Distractibility	0.69	0.64
16 Conscious Attention Control	-0.18	-0.25
17 Emotional Distractibility	0.73	0.72
18 Emotional Perseverance Inhibition (Fear of Failure)	0.53	0.48
19 Implicit Attention Control	-0.64	-0.59
20 Lack of Energy	0.68	0.65
21 Planning	-0.51	-0.49
22 Positive Goal Fantasy	-0.26	-0.29
23 Strategic Intention Control	-0.22	-0.22


*With a sample size of 7400 (i.e., Study 2- Full), all correlations ±0.03 or greater significant at *p* < 0.01. With a sample size of 2435 (i.e., Study 2- Student), all correlations ±0.06 or greater significant at *p* < 0.01.*

Hypothesis 3 states that there is a positive relationship between temptation proximity and procrastination. As seen in **Table 5**, the relationship between these variables is even stronger in the large sample (i.e., *r* = 0.38 versus *r* = 0.53). Previously, our *post hoc* analyses indicated that temptation reflects proximity to environmental tempting factors as well as susceptibility to temptation (an individual difference variable). Confirming this, we subjected this model to a regression analysis with temptation and distractibility (defined by the two VCI subscales *Emotional* and *Attentional Distractibility* scales) as predictors for procrastination. In this model, temptation as well as the two VCI subscales were significantly related to procrastination, temptation less so, β = 0.11, compared to emotional and attentional distractibility, β = 0.46 and β = 0.25, respectively. Hence, individual distractibility does appear to exacerbate frequency of temptations. This also addresses Hypothesis 7, which states that temptation attention control will show a negative relationship with procrastination. Temptation attention can be operationalized through these two VCI subscales, as both deal with the degree people are able to suppress other needs and desires. As seen in **Table 5**, both these facets of temptation attention control are strongly related to procrastination (i.e., *r* = 0.73 and 0.69).

Hypothesis 4 predicts that there will be a negative relationship between energy level and procrastination. As seen in **Table 5**, the VCI’s Lack of Energy scale shared a strong, significant relationship with the procrastination index (i.e., *r* = 0.68). Hypothesis 5 states that the use of routines and habits will show a negative relationship with procrastination. Automaticity was assessed at a trait level with VCI’s Planning, which demonstrated a strong, negative relationship with procrastination (i.e., *r* = -0.51). Hypothesis 6 stating that stimulus control shows a negative relationship with procrastination, was tested with VCI’s Strategic Intention Control subscale, demonstrating a correlation of -0.22.

Hypotheses 8 examines goal attention control, with hypothesis 8a predicting that there will be a negative relationship between goal attention control and procrastination. As in Study 1, goal attention control was measured by two VCI scales, Conscious Attention Control and Implicit Attention Control. The first shared a weak relationship with procrastination, *r* = -0.18 in the full sample and *r* = -0.25 in the student subsample. As in Study 1, Implicit Attention Control, correlated strongly with trait procrastination, *r* = -0.63. Including Conscious Attention Control, Intention Monitoring as well as Implicit Attention Control as predictors for procrastination, the effect of Implicit Attention Control was high, β = 0.54, *p* = 0.00 compared to both Conscious Attention Control (β = 0.05, *p* = 0.00) and Intention Monitoring (β = 0.08, *p* = 0.00). These results again indicate that attempting to focus one’s attention seems to be overridden by automatic and disruptive implicit attentional processes.

Hypothesis 8b focuses on the role of fantasy in reducing procrastination. The VCI’s Positive Goal Fantasy was, as in Study 1, not significantly correlated with dispositional procrastination. To assess the hypothesized interaction effect between Positive Goal Fantasy and Implicit Attention Control, as per Study 1, we subjected this model to regression. Here, a significant effect of Implicit Attention Control was again observed, β = 0.54, *p* = 0.00, whereas the effects of PGF and the PGF-IAC interaction were non-significant. This duplicates Study 1’s finding that the interaction is not significant at a trait procrastination level.

Finally, Hypothesis 9 predicted that when fear of failure is self-reported as reducing motivation, its connection to procrastination should be fully accounted for by impulsiveness and/or lack of energy. As per **Table 5**, VCI’s Emotional Perseverance Inhibition scale, measuring fear of failure, demonstrated a strong relationship with trait procrastination (i.e., *r* = 0.53). Though a measure of impulsiveness was not included in Study 2, as in Study 1, Lack of Energy still largely mediated fear of failure’s relationship with procrastination. Including Lack of Energy as a mediator, the direct relationship between fear of failure and procrastination reduced from β = 0.41 (*p* < 0.001) to β = 0.14 (*p* < 0.001), with the indirect effect via Lack of Energy demonstrating a stronger direct relationship, β = 0.28, *p* = 0.00. For a summary of hypotheses and results across Study 1 and 2, see **Table 6**.

**Table 6:** Summary of results.

Hypothesis	Summary	Supported	
		
		Study 1	Study 2
**Goal choice and goal pursuit**			
H1a	Goal pursuit follows a hyperbolic curve	Yes	NA
H1b	The degree of curve predicted by procrastination	Yes	NA
H2a	Procrastination → Intention action gap not intention	Yes	NA
H2b	H2a larger for distal than proximal goal	Yes	NA
H3	Temptation proximity → Procrastination	Partial	Yes
**Goal striving**			
H4	Lack of energy → Procrastination	Yes	Yes
H5	Routines → Procrastination	Yes	Yes
H6	Stimulus control → Procrastination	Partial	Yes
H7	Temptation attention control → Procrastination	Yes	Yes
H8a	Goal attention control → Procrastination	Partial	Yes
H8b	Positive goal fantasy interaction → Procrastination	Partial	No
H9	Fear of failure → Procrastination	Partial	Partial


*Partial support reflects that the finding was found with only one criterion or predictor.*

## General Discussion

We examined procrastination across three major goal phases: goal choice, goal pursuit, and goal striving. Across these stages, we generate nine hypotheses, finding the majority of them supported. Starting with goal choice and pursuit, people’s typical temporal trajectories or pacing style not only followed a hyperbolic curve but the steepness of this curve was predicted by self-reported procrastination. The intention-action gap, where people fail to follow up on their original work intentions, also showed a differential effect for procrastinators. Though procrastinators made the same work intentions as others, they were particularly sensitive to how proximal these goals were. They particularly needed them close for them to be effective. Our research indicates that the intention-action gap can be partially explained by susceptibility and proximity to temptation. Because procrastinators are particularly impulsive, they are highly attracted to whatever consequences that are nearby and accessible, whether they be goals or diversions. Notably, these results indicate that temporal motivation theory provides a more accurate account of motivation than goal setting theory, which lacks direction regarding differential effects due to impulsiveness or for procrastinators. The results also emphasize the irrational nature of procrastination, that people put off against their own work intentions and the largest performance correlation was with people’s own standards and aspirations.

During goal striving, temporal motivation theory indicates several self-regulatory techniques that should reduce the amount of irrational delay or procrastination, namely: energy regulation, automaticity, stimulus control, temptation attention control, and goal attention control. All of these, except stimulus control, was found to be substantively associated with reduced procrastination. Notably, both forms of attention control, energy regulation, and automaticity all predicted separately and substantively. Altogether, they accounted for 74% of the variance in procrastination, drastically limiting the amount of unique contribution other self-regulatory skills might provide. Also as expected, there was a significant interaction between positive goal fantasy and attention control, though not with trait procrastination. Though fantasy is an extremely popular folk form of motivation, our research here supports Oettingen’s (2012) findings that it is effective with a form of mental contrasting.

Follow up on this, we examined why fear of failure is sometimes viewed as a source of procrastination (e.g., Pychyl and Flett, 2012), despite the extremely low or even negative correlations between the construct and procrastination. Assessing the degree that people had motivational problems they attributed to fear of failure, temporal motivation theory predicted other motivational factors would account for any relationship with procrastination. That is, despite self-diagnosis that motivational problems were due to anxiety related sources, energy levels and impulsiveness should provide an equally full explanation, which is what we found. After controlling for energy levels, the fear of failure connection entirely (Study 1) or largely (Study 2) disappears. With growing research supporting willpower over anxiety-related explanations of procrastination (e.g., Steel and Klingsieck, 2016), this strongly suggests that while anxiety may be experienced during irrational delays, it is unlikely to be a major causal factor for most people.

### Limitations and Future Research Directions

Roe (2014) believes “concentrating research efforts on a few deliberately chosen jobs and worker population will be of great help in gaining a better understanding of performance and motivational dynamics” (p. 100). We agree and argue that the computerized PSI should be among these venues. As discussed, the PSI venue is the prototype for the more modern MOOCs (Martin, 2012), and they lend themselves toward fulfilling almost every desired design element for motivational research. First, they occur in a naturalistic, everyday setting as opposed to an artificial laboratory setting. Second, they permit a large number of people completing the same task at their own pace. For example, the education company Udacity’s *Introduction to Computer Science* has an enrollment of approximately 300,000. Third, the tasks take an extended period of time, permitting procrastination and the study of long-term goals. Fourth, assessment of observed behavior and individual differences can be readily obtained in this environment, with the observed behaviors especially sensitive to procrastination. As a joint survey by *Scientific American* and *Nature*, a review by Bartholet (2013) emphasizes: “in a MOOC, the basic human tendencies toward procrastination and sloth are stronger than in traditional classes” (p. 73). Combined, these features provide exactly the field research and behavioral measurements that make influential and powerful findings, despite the documented sharp decline in actual use (Baumeister et al., 2007; Cialdini, 2009). Overall, the opportunities and features offered by MOOCs can enable the rapid and deep examination of self-regulation. Though further validation of our behavioral measure is needed, potentially findings established in this venue should readily generalize to analogous arenas of performance where there are: (1) distant deadlines, (2) substantial autonomy, and (3) ready access to distractions. For example, labor arbitrators, a job that shares these three characteristics, suffers substantively from procrastination and it is a major determinant of their performance (Taras et al., 2015).

Once this venue is more broadly adopted, we recommend several modifications and extensions to our basic research design. To begin with, Study 1 focused on behavior in one psychology course. Being in a realistic environment, multiple goal pursuits was occurring, though we measured advancement in one area exclusively. If goal pursuit and striving could be measured across several courses at once, especially if they are still constrained to a semester format, it should provide an even richer understanding of motivation. Supplement these behavioral observations with skill assessment and other forms of self-reports, we would have a detailed understanding of why people choose to work, how intensely and for how long.

Second, our study was not experimental in nature and interpretation may be affected by unmeasured third variables. To rectify this, we can start to incorporate experimental designs in this framework, looking for ways to improve motivation. This is especially important for the MOOC environment, which is rife with procrastination and failure to perform, including large numbers of dropouts. This venue could readily incorporate experimental testing, which provides more definitive tests than the correlational research primarily done in this area. An experimental research where people are taught the *best ways*, not just the typical ways, to keep distractions hidden might give improved results. More broadly, the treatment arsenal of the motivational field has yet to progress much beyond assessing individual treatment effect. Though some promising combinations of techniques are emerging, such mental contrasting implementation intentions (Gollwitzer and Oettingen, 2012), these are very much the exception. Our results suggest that self-regulatory mechanisms can be additive and a comprehensive procrastination treatment program should seek to address several components simultaneously (e.g., attentional control, routine building).

As a final future direction, interaction effects between the individual and the treatment should be explored. The same intervention is unlikely to be equally effective to all, but we have little idea presently about who would benefit from what. Given that a finite number of substantive and separate determinants of procrastination are emerging, we should, as Gröpel and Steel (2008) note “move beyond motivational main effects and toward customizing interventions to the individual” (p. 410). The results here point the way toward constructing a comprehensive diagnostic of susceptibilities and skills that identify what interventions are likely to be most promising. In terms of minimizing procrastination, one size does not fit all because individuals are likely to have idiosyncratic holes in their motivational repertoires. For example, consistent with the results we obtained here, we expect that goals should be proximally fine-tuned by how impulsive people are, with shorter delays for the more impulsive.

As a final limitation, in a longitudinal study such as this one with multiple batteries, when to administer measures become an issue. Scales administered closer in time together tend to have higher correlations than those further apart. Also, consistent with the concept of traitedness, you tend to get a better measure of a construct when people have a chance to improve self-knowledge and reflect, such as at course end. On the other hand, this potentially synchronizes state measures with trait, such as potentially increasing the correlation between course-specific or observed procrastination with our trait measure. While our pattern of results remained robust, the trait effect sizes can shift depending on when measurement occurred.

## Conclusion

As Heath (2014) reviews, one among a multitude of societal observers, technological advances are speeding the delivery mechanisms for many of our needs. Unfortunately, these mechanisms tend to favor options that satisfy our needs only in the short term and at considerable long-term cost. The result is that more satisfying ventures are put aside in favor of these *shallow* but more immediate options. Clark (1997), in earlier examination, ominously extrapolating this trend to a *dystopia*, with Carr (2010) concluding that this is increasingly coming about. By constantly surrounding ourselves with easily available but inferior options, we have done ourselves a disservice.

Consequently, we need better theories for making sense of self-regulatory failure and better venues to test them. Such a pairing can make the entire motivation field more epistemologically tractable, increasing our pace of scientific progress, and directing us toward effective diagnoses and treatments aimed at both the individual and the environment. To combat procrastination and other forms of irrational choice, we will often need to implement self-regulatory mechanisms to guide our decision-making. By harnessing our capacity to self-correct, we can prepare for our inevitable fallibility.

## Author Contributions

PS designed, implemented, conducted the analyses for Study 1 and collected the data for Study 2, as well writing the first draft. FS conducted the analyses for study 2 and wrote the analyses. TT helped edit, source and proofread the paper. TB was instrumental in enabling the data for study, helped collect the data for study 1 and provided design advice.

## Conflict of Interest Statement

The authors declare that the research was conducted in the absence of any commercial or financial relationships that could be construed as a potential conflict of interest.
